# Cryptosporidiosis diagnosed using metagenomic next-generation sequencing in a healthy child admitted to pediatric intensive care unit: a case report

**DOI:** 10.3389/fcimb.2023.1269963

**Published:** 2023-10-27

**Authors:** Xiaoyi Liu, Jing Wang, Jun Liu, Xuming Li, Yuanlin Guan, Suyun Qian, Xinlei Jia

**Affiliations:** ^1^ Pediatric Intensive Care Unit, Beijing Children’s Hospital, Capital Medical University, Beijing, China; ^2^ Department of Scientific Affairs, Hugobiotech Co., Ltd., Beijing, China

**Keywords:** *Cryptosporidium parvum*, mNGS, healthy boy, PICU, pancreatitis

## Abstract

**Background:**

*Cryptosporidium* infections in humans typically result in symptoms such as abdominal pain and diarrhea. When the diarrhea is severe, it can cause serious complications and even be life-threatening, especially in patients with compromised immune systems.

**Case presentation:**

Here, we reported the use of metagenomic next-generation sequencing (mNGS) to assist in the diagnosis and treatment of a 10-year-old boy with severe *Cryptosporidium* infection. Despite the absence of any history of immunocompromise, the infection still resulted in severe symptoms, including shock, as well as damage to his pancreas and kidneys. The mNGS tests detected the presence of *Cryptosporidium parvum* when conventional methods failed. The patient received anti-parasite treatment along with supportive care to manage the condition. With disease surveillance based on regular clinical tests and sequential mNGS tests, the child recovered from the severe conditions.

**Conclusion:**

Our study emphasized the importance of recognizing the potential severity of *Cryptosporidium* infection, even among individuals with normal immune systems. Timely diagnosis and ongoing monitoring are essential for patient prognosis.

## Introduction

Cryptosporidiosis, caused by *Cryptosporidium* spp., is a parasitic disease that typically infects the epithelial cells of the digestive tract in both humans and animals, leading to symptoms like abdominal pain and diarrhea. The first documented case of human self-limited enteritis caused by *Cryptosporidium* infection was reported in 1976 (Nime et al., 1976). Since then, *Cryptosporidium* infection has been widely reported in over 90 countries and regions worldwide (Liu et al., 2020), with *Cryptosporidium hominis* and *Cryptosporidium parvum* being responsible for more than 90% of human *Cryptosporidium* infections (Feng et al., 2018). Patients with compromised immune systems who are infected with *Cryptosporidium* may suffer from severe gastroenteritis (Hassanein et al., 2012; Gerace et al., 2019), resulting in copious amounts of watery diarrhea that can cause significant fluid loss and life-threatening complications. Conversely, individuals with normal immune function typically experience asymptomatic infection or mild self-limiting diarrhea as their primary clinical symptom (Ryan et al., 2014). Here, we present a rare case of *C. parvum* infection in a healthy child that resulted in severe dehydration, hypovolemic shock, rhabdomyolysis, acute kidney injury, and pancreatitis due to the presence of massive watery stools and vomiting.

## Case presentation

**Figure 1:**
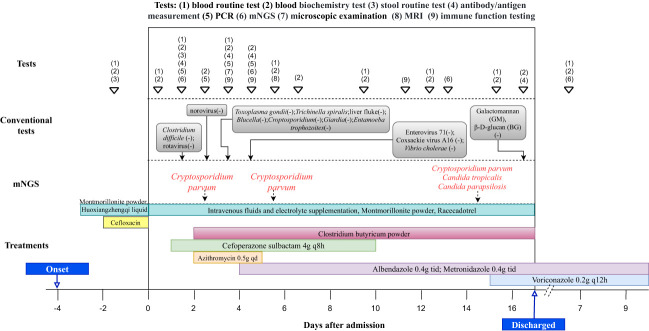
The clinical tests and treatment timeline of the patient.

We reported a case of a ten-year-old boy from Zhangjiakou City in Hebei Province, who presented with persistent symptoms including nausea, abdominal pain, vomiting and severe diarrhea, accompanied by intermittent fever. The child was firstly admitted to a local hospital, where routine examination revealed elevated levels of multiple indicators, as well as the presence of blood and white blood cells in his stool (**Table 1**). Initial treatment involved cefloxacin administration and fluid rehydration (**Figure 1**), but his symptoms did not improve. On the fourth day after the onset of symptoms, he exhibited a decreased level of consciousness, prompting his transfer to the Pediatric Intensive Care Unit (PICU) at Beijing Children’s Hospital Affiliated with Capital Medical University.

**Table 1:** Laboratory test results of peripheral blood during hospitalization.

Tests	2 days before admission (in local hospital)	Day 1	Day 2	Day 3	Day 4	Day 5	Day 6	Day 7	Day 10	Day 12	Day 13	Day 14	Day 16	Day 17	Reference range
Blood routine tests															
WBC (10^9/L)	12.9	18.5	14.2	/	10.4	/	8.3	/	11.5	/	9.9	/	10.1	/	4.3~11.3
PLT (10^9/L)	474	448	300	/	279	/	215	/	560	/	568	/	492	/	167~453
PCT (ng/mL)	4.0	6.1	1.6	/	0.6	/	0.2	/	/	/	/	/	/	/	<0.5
CRP (mg/L)	26.6	13	5	/	5	/	4	/	<0.5	/	<0.5	/	1	/	<8
Blood biochemistry tests															
Hemoglobin (g/L)	169	171	118	/	116	/	111	/	120	/	119	/	123		118~156
Urea (mmol/L)	14.9	23.1	13.8	8.6	5.5	3.0	1.7	1.4	2.2	/	3.4	/	4	4.4	2.7~7
Creatinine (umol/L)	228.2	236.6	121.3	72.7	61.1	58.9	54.9	50.4	49.6	/	47.3	/	48.2	47.3	27~66
ALT(U/L)	/	58.6	52.7	31.5	31.5	33.9	/	35.1	43	/	28.6	/	39	33	7~30
CK(U/L)	562	1327	1379	1444	699	535	/	449	112	/	64	/	54	53	25~200
MB(ng/mL)	757.7	/	/	348.1	/	173.6	108.7	/	/	/	/	/	/	/	0~140
LPS(U/L)	/	98.4	209.5	/	572.5	185	78.1	85.8	212.6	/	205.4	/	222.1	/	0~39
PAMY(U/L)	/	46	60	/	161	77	43	50	106	/	90	/	88	/	17~115
Stool routine tests															
WBC(/HPF)	0~2	/	8	/	/	/	/	/	/	/	/	/	/	/	0
RBC(/HPF)	1~3	/	2-3	/	/	/	/	/	/	/	/	/	/	/	0
Others															
Stool volume(mL)	3~4 times/day	200	696	400	1878	3096	1601	583	0	normal stool	0	normal stool	0	0	
Temperature(°C)	39.2	38	37.5	37.8	37.3	37.5	37.6	36.9	36.8	/	36.8	/	36.8	36.8	36.1~37

WBC, white blood cell; PLT, platelet; PCT, procalcitonin; CRP, C-reactive protein; ALT, alanine aminotransferase; CK, creatine kinase; MB, myoglobin; LPS, lipopolysaccharide; PAMY, pancreatic amylase; RBC, red blood cell.

Upon admission to the PICU, the child had a fever of 38°C and presented with intermittent episodes of confusion, disorganized speech and shock. His physical examination revealed a heart rate of 138 beats per minute, respiratory rate of 26 breaths per minute, and blood pressure of 134/90 mmHg. Clinical signs of dehydration were observed, including dry and less elastic skin, dry lips, as well as sunken eye sockets. His extremities exhibited coldness, and the capillary refill time (CRT) was measured at 4 seconds. Routine blood tests showed leukocytosis (18.5×10^9/^L) and thrombocytosis (448×10^9/^L), as well as elevated levels of C-reactive protein (CRP, 13 mg/L), hemoglobin (171 g/L) and procalcitonin (6.1 ng/mL). Blood biochemical results indicated remarkably high levels of urea (23.1 mmol/L), creatinine (236.6 umol/L), creatine kinase (1,327 U/L) (**Table 1**). Subsequent stool test results revealed 8 white blood cells per high power field.

There were no obvious abnormalities in the patient’s immunoglobulin and lymphocyte counts and proportions. Antishock therapy was administered to him via intravenous fluids and electrolyte supplementation, resulting in the correction of his shock within 6 hours of admission. In view of the elevated levels of creatine kinase and serum myoglobin, a diagnose of rhabdomyolysis was made and the child was treated with hydration and alkalization. Due to severe dehydration caused by vomiting and diarrhea, he received a fluid supplement. In addition, racecadotrel was administered orally to reduce intestinal fluid secretion, montmorillonite powder was given orally to protect the intestinal mucosa, and probiotics were prescribed orally to regulate the intestinal flora. Infectious diarrhea was highly suspected, and cefoperazone sulbactam (4g q8h) was given as empiric therapy (**Figure 1**). The child’s pancreatic enzyme levels were observed to be elevated on day 4 after admission, and subsequent pancreatic magnetic resonance imaging (MRI) indicated abnormalities in pancreatic morphology and filling. This led to a diagnosis of pancreatitis, and somatostatin was administered to inhibit the secretion of pancreatic enzymes.

**Figure 2:**
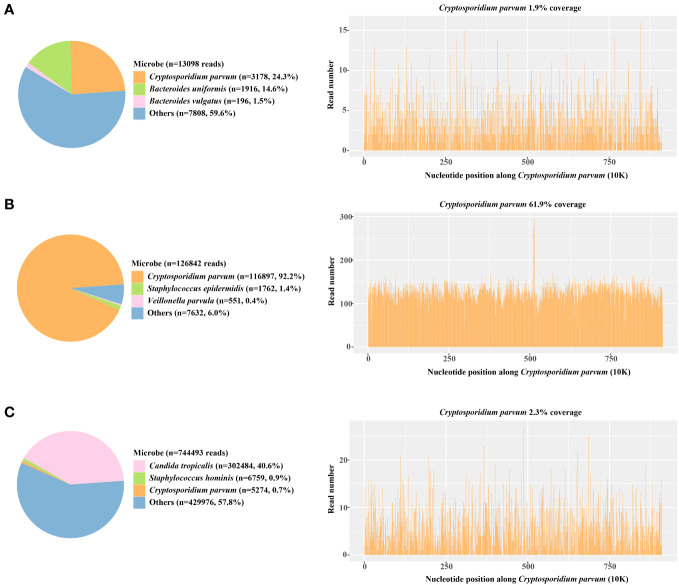
The proportion of detected *Cryptosporidium parvum* sequences in the total microbial sequences and the genome coverage **(A)** Day 3; blood samples **(B)** Day 6; anal swabs **(C)** Day 15; anal swabs.

The antigen test for rotavirus was negative, and negative PCR results ruled out the presence of norovirus and *Clostridium difficile*. Metagenomic next-generation sequencing (mNGS) tests were performed with the consent of the family. Given the possibility of a systemic infection, the whole blood samples of the child were collected on Day 2 after admission, and sent for nucleic acid extraction, library construction and high-throughput sequencing (Hugo-Biotech Company, Beijing). After the sequence of human genome was excluded, mNGS identified 3,178 specific reads belong to *C. parvum*, accounting for 1.9% of nucleotide sequence coverage (**Figure 2A**). The test results were obtained on Day 3, and the patient was promptly initiated on intravenous infusion of azithromycin (0.5g qd) for anti-parasitic treatment. After 2 days, the medication was transitioned to oral administration of metronidazole (0.4g tid) and albendazole (0.4g tid) tablets. During this period, the patient’s peripheral blood cultures were negative. Additionally, tests for IgG antibodies against various pathogens, including *Toxoplasma gondii*、*Trichinella spiralis*、Dengue fever and liver fluke, were negative. The *Brucella* tiger red test was also negative, as were tests for *Cryptosporidium* and *Giardia* antigens (the colloidal gold method) in stool samples. Furthermore, PCR results of *Entamoeba trophozoites* and cysts, Enterovirus 71, Coxsackie virus A16, and *Vibrio cholerae* slide agglutination test were all negative. Microscopy examination did not reveal any fungi or parasites.

On Day 5 after admission, anal swabs of the child and his parents were collected for further detection using mNGS. The results of child’s tests showed that 92.2% (116,897) of pathogenic reads corresponded to *C. parvum* with a coverage of 61.9%, and neither parent was detected with any pathogen (**Figure 2B**). On day 10, the child’s temperature and inflammatory markers returned to normal levels, and cefoperazone sulbactam was discontinued. Additionally, his vomiting and gastrointestinal symptoms were relieved, and the levels of pancreatic enzymes, creatinine and creatine kinase were lower than before. On Day 15, mNGS identified 5,274 specific reads of *C. parvum* in the child’s anal swabs samples, indicating a decrease in pathogen nucleic acid load. However, *Candida tropicalis* and *Candida parapsilosis* (302,484 and 5,320 specific reads) were also detected in this test (**Figure 2C**). This was considered to be caused by an imbalance in intestinal flora, and oral voriconazole (0.2g q12h) was given to prevent opportunistic infections. On Day 17, the patient’s temperature was normal without vomiting or diarrhea, and he was discharged. After discharge, he continued to take albendazole, metronidazole, and voriconazole tablets orally for 10 days, and no fever, diarrhea, or vomiting recurred. On the 7th day after discharged, his blood routine test, liver and kidney function, and pancreatic enzyme levels returned to normal, and re-examination using mNGS showed no presence of pathogens.

## Discussion

*Cryptosporidium* oocysts are common in the environment, and in China, the primary risk factors for infection include consumption of contaminated water or food, contact with infected animals, and seasonal outbreaks of *Cryptosporidium* (usually during summer or autumn) (Feng and Xiao, 2017). The faecal-oral route is the primary mode of transmission for this parasite. The child in this report lived in Hebei Province, where cryptosporidiosis has rarely been reported. He fell ill in the summer, and denied any exposure with domestic animals. He had spent a short time in a rural village in Hebei Province and drank from a well, but he lived and ate with his parents, who were not infected with *Cryptosporidium*. Despite having no history of immune dysfunction, the child experienced severe symptoms including shock, and damage to the pancreas and kidneys after contracting the infection.

*Cryptosporidium* is known to be able to cause severe gastroenteritis (Bouzid et al., 2013; Gerace et al., 2019), with immunocompromised individuals experiencing more serious complications. The development of kidney injury in *Cryptosporidium* infection may be explained by the mechanism of direct cytopathic injury as observed in cases of nematode infections (Prasad and Patel, 2018). Previous reports have described pancreatitis associated with *Cryptosporidium* in both immunocompromised and immunocompetent patients (Miller et al., 1992; Norby et al., 1998). The pathogenesis of parasite-induced pancreatitis may involve worm obstruction of the pancreatic duct, the common bile duct, or the formation of abscesses (Rawla et al., 2017). A comprehensive understanding of the inflammatory syndromes and complications associated with *Cryptosporidium* infection is crucial for guiding appropriate disease management. Furthermore, our case also restated that *Cryptosporidium* can pose a serious threat even to healthy individuals, emphasizing the need for vigilance towards cryptosporidiosis.

Serology tests for *Cryptosporidium* usually exhibits high sensitivity and specificity (Ungar, 1990; Agnamey et al., 2011; El-Moamly and El-Sweify, 2012). However, false negative results may occur when the concentration of oocysts in samples is low (El-Moamly and El-Sweify, 2012). In this study, the intermittent shedding pattern observable in *C. parvum* infection (Jokipii and Jokipii, 1986; Chappell et al., 1996) may account for the initial negative findings obtained by immunochromatographic assay. PCR testing has been shown to have excellent performance for *Cryptosporidium* diagnosis (Helmy et al., 2014), and qPCR can serve as an accurate quantification method for assessing pathogenic load during disease progression (Clementi et al., 1995; Caldas et al., 2012). Nevertheless, designing primers for PCR requires expertise and can be time-consuming. Multiplex PCR panels targeted towards enteric pathogens, such as FilmArray (Golan Shaposhnik et al., 2019) and TaqMan Array Card (Liu et al., 2013), have been increasingly used in the early workup of a severely ill patient with diarrhea. However, these panels are not currently widely accessible in Chinese hospitals. Hence, in this case, mNGS was selected for pathogen detection to avoid diagnostic delays.

mNGS has emerged as a crucial technique for identifying unexplained infections, with the potential to detect all pathogen sequences in a single test without requiring specific amplification or primers. Its use is increasingly widespread in clinical settings to diagnose various infectious diseases (Chiu and Miller, 2019; Gu et al., 2021). In this study, mNGS was able to rapidly detect *Cryptosporidium* in the child’s blood samples within two days, enabling adjustments in the clinical treatment plan based on the detection results. This highlights the potential of mNGS to provide timely and accurate diagnosis. Additionally, semi-quantitative mNGS reads have been reported to reflect disease progression and therapeutic effectiveness (Ai et al., 2018; Zhang et al., 2020; Chen et al., 2022). Shedding of *Cryptosporidium* is known to be prolonged after recovering from initial symptomatic illness (Johansen et al., 2022). The presence of *Cryptosporidium* reads identified by mNGS on Day 15 in this case provides additional confirmation of this phenomenon.

The application of mNGS in pathogen detection also has certain limitations. In comparison to multiplex PCR panels, mNGS incurs higher costs and longer turnaround time (Chiu and Miller, 2019; Ramachandran and Wilson, 2020). The abundance of data generated by mNGS necessitates intricate bioinformatics analysis and interpretation, demanding specialized personnel and advanced computational resources. Additionally, the heightened sensitivity of mNGS technology may result in the introduction of noise and false-positive outcomes (Jing et al., 2021). This could arise from exogenous DNA contamination, cross-contamination during sample handling, or errors during sequencing procedures (Chiu and Miller, 2019). These constraints presently hinder mNGS from emerging as a forefront diagnostic tool in clinical settings, but ongoing technological advancements are continuously improving its potential. Additionally, although not utilized in this particular study, it is crucial to emphasize the important role of multiplex PCR panels in the diagnosis and surveillance of *Cryptosporidium* infection.

At present, there is currently no specific drug treatment for cryptosporidiosis. Nitazoxanide has been proven effective and is the only drug approved by the Food and Drug Administration to treat cryptosporidiosis, but it is less effective for AIDS patients and malnourished children (Amadi et al., 2002; White, 2004). Azithromycin (Dionisio et al., 1998), paromomycin (Marshall and Flanigan, 1992), and acetylated spiramycin (Huang et al., 2015) have also shown some efficacy. Due to the unavailability of nitazoxanide in China, azithromycin was used as an antiparasitic for three days. Given the rapid improvement in inflammation and pancreatitis markers by day 4, it is likely that azithromycin had a beneficial effect on this patient. However, despite this improvement, the patient’s gastrointestinal symptoms did not alleviate, and the volume of diarrhea continued to increase. Unfortunately, we were unable to add paromomycin and acetylated spiramycin to the treatment regimen due to the unavailability of these medications within a short timeframe.

After consulting with the Institute of Tropical Medicine at Friendship Hospital Beijing, albendazole and metronidazole were prescribed. Although this treatment strategy is typically used for *Giardia* infection (Morch et al., 2008; Rossignol, 2010), the clinical experience of the institute suggested that it can be effective against *Cryptosporidium* infection as well. Efficacy of metronidazole and albendazole in treating *Cryptosporidium* infection has been reported in mice (Fayer and Fetterer, 1995; Abouel-Nour et al., 2016), leading us to hypothesize their potential benefits in this case. It is worth noting that the child’s normal immune system likely contributed to the favorable outcome once adequate hydration was achieved. Additionally, probiotics have been shown to prevent further intestinal damage in children with diarrhea caused by *Cryptosporidium* and can affect intestinal function, immune response, and clinical outcomes (Pickerd and Tuthill, 2004; Sindhu et al., 2014).

During the child’s anti-parasitic treatment, a regimen of cefoperazone sulbactam was also administered. Although no bacterial pathogens were identified, we cannot rule out the possibility of bacterial infection in this case. Upon admission, the patient’s procalcitonin and CRP levels were significantly elevated. Early antibiotic treatment (cefloxacin) prior to transfer to our hospital may have resulted in the omission of other potential bacterial co-pathogens, which could have aggravated the condition and contributed to pancreatitis development. The parasite-bacteria interaction within the human host often have negative consequences, and reports have suggested that superimposed bacterial infections in parasitic diseases can lead to pancreatitis (Singh et al., 2004; Ashour and Othman, 2020). Previous research has demonstrated the superiority of mNGS over traditional tests in patients who have received empirical treatment. However, the efficacy of mNGS may decline with prolonged medication (Zhang et al., 2020). In addition, it is possible that the blood sample used for the initial mNGS test had a relatively low bacterial load, as it was not directly collected from the infection site, thus potentially affecting the detection sensitivity.

## Conclusion

By utilizing mNGS, we identified *Cryptosporidium parvum* in a healthy boy, which resulted in a life-threatening condition. Our study underscored the potential for severe consequences associated with *Cryptosporidium* infection in immunocompetent patients, emphasizing the importance of timely diagnosis and regular monitoring of disease progression in the management of cryptosporidiosis.

## Data availability statement

The datasets presented in this study can be found in online repositories. The names of the repository/repositories and accession number(s) can be found below: https://ngdc.cncb.ac.cn/?lang=zh, PRJCA018717.

## Ethics statement

The studies involving humans were approved by the Clinical Research Ethics Committee of Beijing Children’s Hospital, Capital Medical University. The studies were conducted in accordance with the local legislation and institutional requirements. Written informed consent for participation in this study was provided by the participants’ legal guardians/next of kin. Written informed consent was obtained from the minor(s)’ legal guardian/next of kin for the publication of any potentially identifiable images or data included in this article. Written informed consent was obtained from the participant/patient(s) for the publication of this case report.

## Author contributions

XL: Formal Analysis, Investigation, Writing – original draft. JW: Formal Analysis, Investigation, Writing – original draft. JL: Investigation, Writing – original draft. XML: Data curation, Methodology, Writing – original draft. YG: Methodology, Supervision, Writing – review & editing. SQ: Conceptualization, Supervision, Validation, Writing – review & editing. XJ: Conceptualization, Supervision, Validation, Writing – review & editing.
